# TMS-EEG Research to Elucidate the Pathophysiological Neural Bases in Patients with Schizophrenia: A Systematic Review

**DOI:** 10.3390/jpm11050388

**Published:** 2021-05-10

**Authors:** Xuemei Li, Shiori Honda, Shinichiro Nakajima, Masataka Wada, Kazunari Yoshida, Zafiris J. Daskalakis, Masaru Mimura, Yoshihiro Noda

**Affiliations:** 1 Department of Neuropsychiatry, Keio University School of Medicine, Tokyo 160-8582, Japan; lixmovo@gmail.com (X.L.); shiori.0913.honda@keio.jp (S.H.); masa.wada0622@gmail.com (M.W.); mimura@a7.keio.jp (M.M.); 2 Pharmacogenetics Research Clinic, Centre for Addiction and Mental Health, Toronto, ON M5T 1R8, Canada; kadu.yoshida@gmail.com; 3 Department of Psychiatry, UC San Diego Health, San Diego, CA 92093, USA; zdaskalakis@health.ucsd.edu

**Keywords:** cortical excitation, cortical inhibition, electroencephalography, schizophrenia, transcranial magnetic stimulation, TMS-evoked potentials

## Abstract

Schizophrenia (SCZ) is a serious mental disorder, and its pathogenesis is complex. Recently, the glutamate hypothesis and the excitatory/inhibitory (E/I) imbalance hypothesis have been proposed as new pathological hypotheses for SCZ. Combined transcranial magnetic stimulation (TMS) and electroencephalography (EEG) is a non-invasive novel method that enables us to investigate the cortical activity in humans, and this modality is a suitable approach to evaluate these hypotheses. In this study, we systematically reviewed TMS-EEG studies that investigated the cortical dysfunction of SCZ to examine the emerging hypotheses for SCZ. The following search terms were set in this systematic review: (TMS or ‘transcranial magnetic stimulation’) and (EEG or electroencephalog*) and (schizophrenia). We inspected the articles written in English that examined humans and were published by March 2020 via MEDLINE, Embase, PsycINFO, and PubMed. The initial search generated 379 studies, and 14 articles were finally identified. The current review noted that patients with SCZ demonstrated the E/I deficits in the prefrontal cortex, whose dysfunctions were also associated with cognitive impairment and clinical severity. Moreover, TMS-induced gamma activity in the prefrontal cortex was related to positive symptoms, while theta/delta band activities were associated with negative symptoms in SCZ. Thus, this systematic review discusses aspects of the pathophysiological neural basis of SCZ that are not explained by the traditional dopamine hypothesis exclusively, based on the findings of previous TMS-EEG research, mainly in terms of the E/I imbalance hypothesis. In conclusion, TMS-EEG neurophysiology can be applied to establish objective biomarkers for better diagnosis as well as to develop new therapeutic strategies for patients with SCZ.

## 1. Introduction

### 1.1. Overview of Schizophrenia

Schizophrenia (SCZ) is a serious mental disorder that affects approximately 1% of the population worldwide. SCZ is characterized by positive symptoms such as hallucinations/delusions, negative symptoms such as reduced motivation and blunted affect, and cognitive symptoms, which are considered core features of the illness [1,2,3]. As one of hypotheses to explain the pathophysiology of SCZ, the dopamine hypothesis has been classically proposed [4]; however, the glutamate hypothesis, gamma-aminobutyric acid (GABA) hypothesis, cholinergic hypothesis, and especially the excitatory/inhibitory (E/I) imbalance hypothesis have also been attracting attention in recent years because some pathologies in this disorder cannot be explained by the dopamine hypotheses alone [3,5,6].

Ketamine and phencyclidine, which were developed as dissociative anesthetics in the 1970s, are known to cause schizophrenia-like symptoms such as negative symptoms and cognitive symptoms as well as psychotic symptoms and thought disorder in healthy subjects. In this context, the dysfunction of glutamate receptors is thought to be particularly relevant to negative symptoms and cognitive impairment in schizophrenia. Thus, dysfunction of N-methyl-D-aspartate (NMDA) receptors has recently been proposed as one of hypotheses to explain the pathogenesis of schizophrenia. The glutamate hypothesis in SCZ suggests that the hypofunction of NMDA receptors on interneurons would cause hyperexcitability of the cerebral cortex, leading to symptoms of the disease [7].

Neural computation, the basis for the expression of mental functions, is through balanced E/I, primarily by the glutamatergic system and GABAergic input. Excitation allows neurons to respond to stimuli, while inhibition regulates neuronal selectivity and allows for accurate neural representation [8,9]. Indeed, the E/I balance is necessary for optimal neural signal formation, synchrony, and transmission, which in turn support information processing that drives both simple and complex behaviors. Thus, the E/I imbalance hypothesis has been conceptualized as a pathology caused by an imbalance between glutamatergic and GABAergic inputs and is thought to underlie brain dysfunction in neuropsychiatric disorders, including SCZ [10,11]. Here, a common assumption throughout this hypothesis is that an increase in the E/I ratio, that is, an increase in excitation or a decrease in inhibition, is involved in the expression of psychiatric symptoms. In this context, several lines of evidence indicate that patients with SCZ showed decreased glutamate and GABA levels in the prefrontal cortex (PFC) as measured by proton magnetic resonance spectroscopy (^1^H-MRS) [12,13,14]. However, on the other hand, recent meta-analysis studies have shown inconsistencies in the findings of these MRS findings [15,16]. One way to address these limitations is the development of a combined transcranial magnetic stimulation (TMS) and electroencephalography (EEG) technique that can non-invasively assess the neurophysiological properties of the human cerebral cortex [2,3,17].

### 1.2. Technical Advance of Combined TMS-EEG

TMS was first introduced by Barker et al. in 1985 to investigate the corticospinal circuits applying to the primary motor cortex (M1) in humans [18]. When a single-pulse TMS is administered to M1, motor-evoked potential (MEP) is elicited, and its amplitude is thought to reflect corticospinal excitability. In addition, TMS neurophysiology has recently been combined with EEG to assess E/I profiles in specific cortical areas outside of the motor cortex as well [19,20]. Although most previous TMS studies in patients with SCZ have examined cortical excitability and inhibition in M1, it has been assumed that neurophysiological dysfunction in the PFC, rather than M1, may be relevant in patients with SCZ.

EEG is able to provide a direct measure of the electrical signals generated by neuronal activity and allow us to detect the E/I profiles of network connections [20]. Specifically, high-density electroencephalography (EEG) has a high-temporal resolution as well as better spatial resolution compared to the conventional EEG system due to the increased number of channels such as EEG electrodes with 64 or more channels. Hence, the combination of TMS with EEG recording (TMS-EEG) can be applied to probe and investigate the cortical responses and propagation of TMS-evoked EEG between some specific brain areas [21,22,23]. In particular, since TMS-evoked potentials propagate to brain regions that are anatomically and functionally connected [21,24], simultaneous measurement with EEG, which has high temporal resolution, can accurately detect the temporal pattern of TMS-evoked responses and is likely to identify causal relationships of connectivity between brain regions. In other words, the spatial and temporal patterns of brain responses to TMS can contribute to defining causal relationships in the connections across brain areas.

### 1.3. TMS-EEG Neurophysiology

#### 1.3.1. GABAergic (Short-Interval Intracortical Inhibition: SICI and Long-Interval Intracortical Inhibition: LICI) and Glutamatergic (Intracortical Facilitation: ICF) Neurophysiological Functions

GABA is a critical inhibitory neurotransmitter to modulate cortical excitability and neuroplasticity of the brain. There are two main types of GABA receptors, GABA_A_ and GABA_B_ receptors for mediating cortical inhibition. Specifically, GABA_A_ receptor-mediated cortical inhibitory function can be measured with short-interval intracortical inhibition [25,26], which is a paired-pulse paradigm of TMS that consists of one subthreshold preceding stimulus (conditioning stimulus) and one suprathreshold following stimulus (test stimulus) with an interstimulus interval (ISI) of 1 to 5 ms [27]. The conditioning stimulus activates a low threshold inhibitory network, causing inhibitory postsynaptic potentials to hyperpolarize the neurons when the following test pulse is applied [25,27,28]. In addition, pharmacological studies combined with TMS neurophysiology demonstrated that benzodiazepines (i.e., agonists for the GABA_A_ receptors) increased the SICI [25,29,30,31,32,33,34], suggesting that SICI may be closely related to GABA_A_ receptor-mediated inhibition. Further, other pharmaco-TMS studies indicated that baclofen, specific GABA_B_ receptor agonists, increased the LICI [35,36], suggesting that LICI is mediated through the GABA_B_ receptors. LICI comprises two suprathreshold stimuli with an ISI of 50 to 150 ms. In both SICI and LICI, final outputs of MEP amplitudes or TMS-evoked potential (TEP), which can be measured with EEG, are reduced by the test stimulus. On the other hand, glutamatergic acid is a main excitatory neurotransmitter in the brain and glutamatergic N-methyl-D-aspartate (NMDA) receptor-mediated cortical facilitation can be measured with intracortical facilitation (ICF) paradigm [33]. ICF is a paired-pulse TMS paradigm that uses the same conditioning and test stimulus intensities as SICI while the ISI of ICF is 10 to 15 ms. Other studies also revealed that NMDA receptor antagonists decreased the ICF [36,37], suggesting that ICF may be closely associated with facilitation mediated through glutamatergic NMDA receptors.

#### 1.3.2. Cholinergic (Short-Latency Afferent Inhibition: SAI) Neurophysiological Function

SAI is mainly considered to reflect cholinergic function and is partially mediated by GABA_A_ receptor function [30,31,32]. Regarding SAI, it was increased by donepezil, an acetylcholinesterase inhibitor, while it was reduced by scopolamine, muscarinic acetylcholine receptor antagonists. SAI was also increased by diazepam, the GABA_A_ receptor agonists [32,38,39]. Moreover, previous studies have demonstrated that the SAI is associated with cognitive function, which is mediated through cholinergic function [40,41]. Previous studies have explained that the SAI is associated with cholinergically modulated cognitive function [40,41]. The SAI paradigm consists of a preceding peripheral median nerve stimulus and a subsequent single-pulse TMS. Median nerve stimulation is set at the intensity of three times the sensory thresholds, while TMS is set at the intensity of suprathreshold. The sensory afferent stimulation inhibits MEP or TEP. For the SAI paradigm applied to M1, the latency of somatosensory evoked potential N20 plus 2 ms induced by peripheral median nerve stimulation is used for the ISI between median nerve stimulation and TMS. In contrast, when the SAI is applied to the dorsolateral prefrontal cortex (DLPFC), the ISI of N20 plus 4 ms is used to evaluate the optimal SAI change from the DLPFC [42].

#### 1.3.3. Other Neurophysiological Measures in TMS-EEG (Power, ERSP, Coherence, and Natural Frequency)

EEG power indicates the time-averaged value of wave energy, and it can be calculated according to the frequency bands, from the slow wave delta component to the fast wave gamma component. In SCZ, a decrease in EEG power of theta and gamma bands is indicated, especially in relation to cognitive impairment. Event-related spectral perturbation (ERSP), a generalization of event-related desynchronization, is a measure of the average dynamic change in the amplitude of the broadband EEG frequency spectrum as a function of time relative to an experimental event. In other words, ERSP measures the average time course of relative changes in the spontaneous EEG amplitude spectrum evoked by a series of similar experimental events, in this case TMS stimulation. EEG coherence is a linear synchronization measure between two signals over time recorded at different electrode sites, which is specifically a statistical measure of the average agreement in phase difference weighted by amplitude and is frequency dependent, showing a value from 0 (completely random phase difference) to 1 (perfectly matched phase difference) [43]. Furthermore, natural frequency is obtained by measuring the main frequency associated with direct TMS perturbations to the brain, which is mainly related to frequency tuning in the corticothalamic system. Indeed, the measurement of natural frequencies may provide important information about the properties and state of a particular brain system [44].

### 1.4. Objectives of This Systematic Review

In previous reviews, GABAergic dysfunction as well as gamma oscillatory abnormalities in SCZ have been well described [2,3,45,46,47,48]. However, these reviews did not discuss in detail the neurophysiological indices, including glutamatergic and cholinergic functions in SCZ, from the perspective of neurotransmitters. Thus, the current systematic review aimed to comprehensively assess and summarize the previous TMS-EEG studies on neurophysiological dysfunctions in patients with SCZ, including glutamatergic and cholinergic functions, and to discuss the useful role of combined TMS-EEG that can contribute to elucidating the pathophysiology of SCZ. To this end, we systematically reviewed TMS-EEG studies that compared neurophysiological findings of TMS-EEG studies, such as TMS-evoked potentials, oscillatory activities, and functional connectivity, in the various cortical areas between patients with SCZ and healthy controls (HC). Furthermore, we discussed the importance of TMS-EEG neurophysiology that may lead to a better understanding of the underlying pathophysiology of SCZ as well as the clinical application of this special modality.

## 2. Materials and Methods

### 2.1. Study Search and Selection Strategy

The search was conducted on PubMed, MEDLINE, Embase, and PsycINFO (May 2000 to March 2020) with the following search items: “(Schizophrenia) and (TMS or (transcranial magnetic stimulation)) and (EEG or electroencephalog*) not ((tDCS) or (transcranial direct current stimulation)) not ((ECT) or (electroconvulsive therapy)) not ((DBS) or (deep brain stimulation)) not ((MST) or (magnetic seizure therapy))”. Studies were included if (i) they were written in English; (ii) they compared participants diagnosed with SCZ and HC; and (iii) they measured TMS neurophysiology using combined TMS-EEG. In addition, the following articles were excluded if they were (i) animal model studies; (ii) review articles; or (iii) conference reports. We also excluded clinical studies applying repetitive TMS for treatment, since this review focused primarily on TMS-EEG neurophysiology in patients with SCZ. Then, we reviewed the titles and abstracts of the remaining studies and selected those that utilized TMS-EEG measures to characterize cortical excitability, inhibition, oscillatory activity, or connectivity in patients with SCZ. Next, we read through the full text of the included studies to identify the relevant data.

### 2.2. Data Extraction

Two investigators (X.L. and S.H.) assessed the studies based on the eligibility criteria independently. The following data were extracted from each study: (1) characteristics of participants; (2) parameters and areas of TMS; (3) cognitive/clinical measures; (4) outcome measures; (5) neurophysiological findings; and (6) clinical/cognitive correlations. Then, we summarized the neurophysiological findings from the studies that included HC as a control. Any discrepancies in data extraction were discussed and resolved with the senior author, Y.N.

### 2.3. Outcome Measures

The outcome measures focused on TMS neurophysiology regarding TMS-evoked potentials, functional connectivity, and time-frequency analysis. In this review, M1 and DLPFC were the main areas of interest; however, where TMS neurophysiology studies in other areas have been reported, those areas were also included.

### 2.4. Risk of Bias Assessment

Risk of bias for the included studies was assessed with the Risk of Bias Assessment for Non-randomized Studies tool. The following items were evaluated: participant selection, confounding variables, intervention measurement, blinding of outcome assessment, incomplete outcome data, and selective outcome reporting.

## 3. Results

### 3.1. Characteristics of the Included Studies

**Figure 1:**
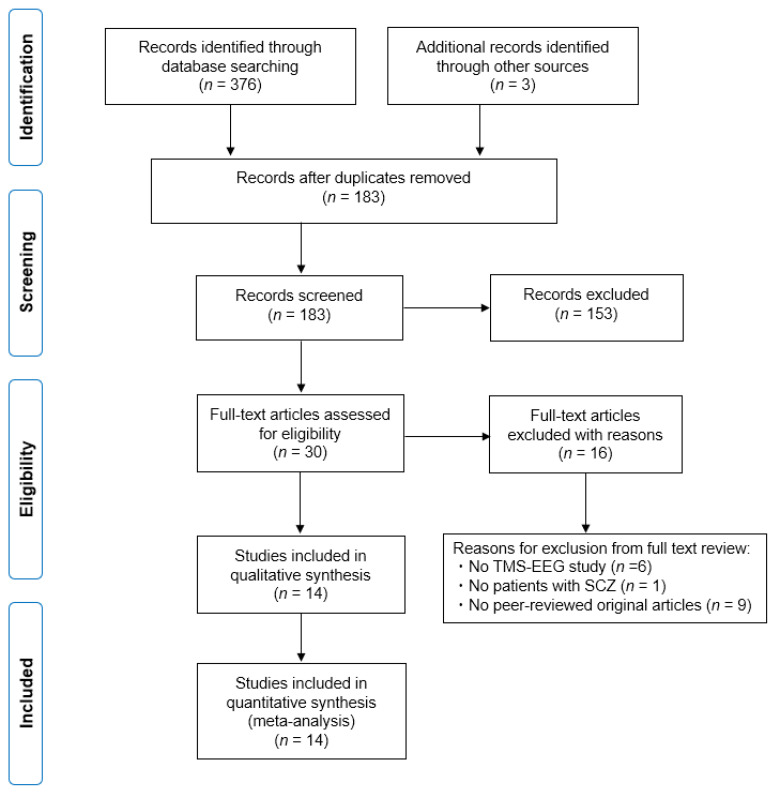
The PRISMA flow diagram.

The initial search generated a total of 376 articles from MEDLINE, Embase, PsycINFO, and PubMed. Additionally, we found another three articles by manual search. Finally, we identified 14 articles. The Preferred Reporting Items for Systematic Reviews and Meta-Analyses (PRISMA) flow diagram is shown in Figure 1.

Figure 1 depicts the flow diagram regarding the information on the different phases in this systematic review, mapping out the number of records identified, included and excluded, and the reasons for exclusions.

In the included studies, regarding the stimulation site, eight studies examined TMS neurophysiology in M1 [41,49,50,51,52,53,54,55], five studies in the premotor cortex [41,49,50,56,57], two studies in the parietal cortex [49,50], eight studies in the DLPFC [41,49,50,54,55,58,59,60], and one study in the other cortical regions [61], respectively. Six studies examined two or more cortical areas in the same study [41,49,50,54,55,60]. In addition, for the stimulation paradigm, nine studies investigated single-pulse TMS including SAI paradigm [41,49,50,51,52,53,56,57,61]. However, there was no study on paired associative stimulation paradigm in patients with SCZ. In contrast, five studies examined paired-pulse TMS including SICI, ICF, and LICI [54,55,58,59,60]. Furthermore, as for the analysis method, 5 studies analyzed TMS-evoked potentials [41,49,57,58,61], 10 studies conducted time-frequency analysis [50,51,52,53,54,55,56,58,59,60], and 4 studies explored functional connectivity [49,54,55,57] from the TMS-EEG data.

### 3.2. Schematic Summary of the TMS-EEG Findings from the DLPFC in Patients with SCZ

**Figure 2:**
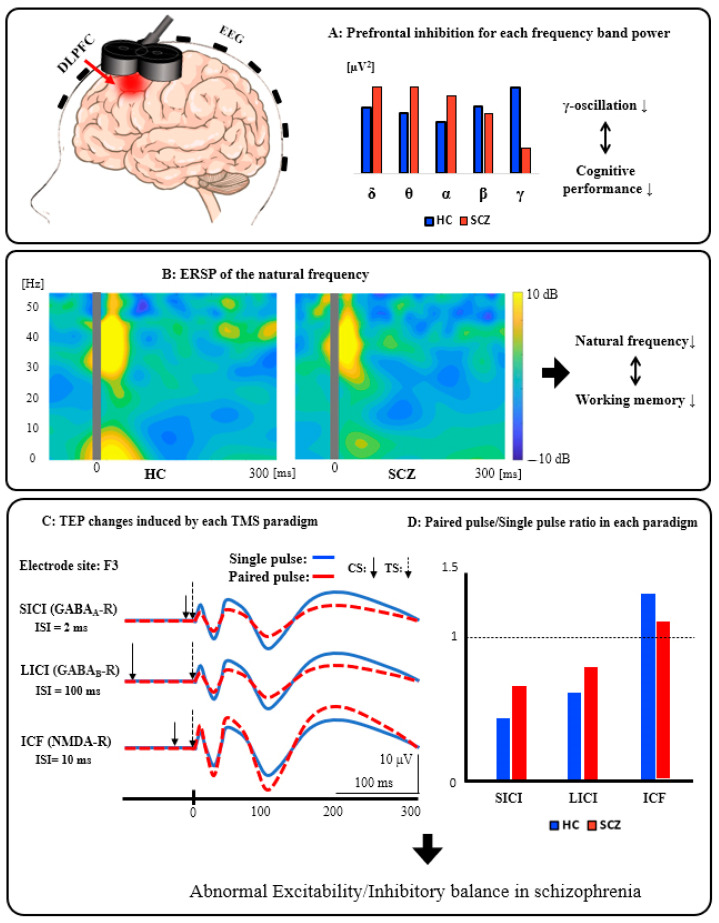
Schematics of combined TMS-EEG neurophysiology from the DLPFC.

We summarized the schematics of the TMS-EEG neurophysiology from the DLPFC in Figure 2.

Figure 2A: Prefrontal inhibition for each frequency band power. Patients with SCZ have significantly reduced activity in the inhibitory gamma band of the prefrontal cortex compared to HC, indicating that it can be associated with cognitive dysfunction. Figure 2B: ERSP of the natural frequency. In patients with SCZ, the natural frequency of the prefrontal cortex is significantly reduced compared to HC, and this reduction can be associated with impaired working memory. Figure 2C: TEP changes induced by each TMS paradigm. These waveforms schematically illustrate the changes in TEP induced by each TMS paradigm (SICI, LICI, and ICF). Figure 2D: Paired pulse/Single pulse ratio in each paradigm. This bar graph is a schematic illustration of the group differences between HC and SCZ in each TMS paradigm. Specifically, based on the results of previous studies, compared with HC, patients with SCZ showed significantly reduced SICI (GABA_A_ receptor-mediated neurophysiological activity), which may be associated with executive dysfunction, as well as significantly reduced LICI (GABA_B_ receptor-mediated neurophysiological activity), which also may be related to working memory deficit. In addition, patients with SCZ had significantly reduced ICF (glutamatergic NMDA receptor-mediated neurophysiological activity) compared with HC, which may be associated with the clinical severity in this disorder. Collectively, it is assumed that excitatory and inhibitory imbalance could be caused by neurophysiological dysfunction, mainly in the prefrontal cortex, as indexed by SICI, LICI, and ICF in patients with SCZ.

### 3.3. TMS-Evoked Potentials (TEP) Analyses

#### 3.3.1. Single-Pulse TMS Paradigm

The results are summarized in Table 1. SCZ had altered functional connectivity [61] and cholinergic dysfunction in the DLPFC compared with HC [41]. Levit-Binnun et al. administered single-pulse TMS over the Cz electrode site and compared the amplitude and latency of TEP between patients with SCZ and HC [61]. After TMS pulse, as a short latency EEG response, HC showed frontal negativity and parietal positivity, while patients with SCZ showed no frontal negativity or greatly reduced parietal positivity. These different patterns of the amplitude and latency of TEPs on topological plots indicate that patients with SCZ have altered functional connectivity between distributed brain areas, which may also cause abnormal cognitive functioning.

SAI: Noda et al. compared SAI in the left M1 and DLPFC between patients with SCZ and HC [41]. They analyzed major TEP components such as N100 and P180 in each condition. They found that patients with SCZ had a significantly smaller modulation of N100 by DLPFC-SAI compared with HC, which was also correlated with executive function as measured by the Trail Making Test. These findings suggest that patients with SCZ may have cholinergic dysfunction in the DLPFC and that this may cause their executive dysfunction.

#### 3.3.2. Paired-Pulse TMS Paradigm

SICI and ICF: The results are shown in Table 1. SCZ showed GABA_A_ receptor-mediated and glutamatergic NMDA receptor-mediated neurophysiological dysfunctions in the DLPFC [58]. Noda et al. investigated SICI and ICF from the DLPFC in patients with SCZ and HC [58]. Amplitudes for each TEP component (i.e., P30, N45, P60, N100, and P180), frequency band powers of TEP, and time-frequency of TEP were separately analyzed for each condition. They found that patients with SCZ showed reduced inhibition in TEP P60 by SICI compared with HC, which was correlated with the longest span of the Letter-Number Span Test in patients with SCZ. On the other hand, compared with HC, patients with SCZ showed reduced facilitation in TEP P60 and N100 by ICF, which correlated with the total score of the Positive and Negative Syndrome Scale (PANSS). This study suggests that GABA_A_ receptor-mediated and glutamatergic NMDA receptor-mediated neurophysiological dysfunctions in the DLPFC may be associated with the underlying pathophysiology of cognitive and clinical symptoms in patients with SCZ.

### 3.4. Time-Frequency Analyses

#### 3.4.1. Single-Pulse TMS Paradigm

The results are summarized in Table 2. Natural frequencies are the major endogenous frequencies that are caused by external perturbations. The human cerebral cortex tends to maintain a natural frequency in each cortical region. Therefore, TMS-EEG can be used to directly stimulate the cerebral cortex and measure its natural frequencies [44]. SCZ showed the remarkable pathophysiological changes in natural frequency [50]. That is, excessive gamma and theta/delta activations by single-pulse TMS over the M1 were observed, which were also associated with clinical symptoms [53]. Ferrarelli et al. applied single-pulse TMS over the M1, DLPFC, and premotor and posterior parietal areas in patients with SCZ and HC [50]. They assessed cognitive function by measuring the accuracy and reaction time for the word memory and facial memory tasks. Intertrial coherence and event-related spectral perturbation (ERSP) were analyzed for the time-frequency domain. When single-pulse TMS was applied to each cortex, natural frequency induced by TMS became faster in the order of the parietal cortex, M1, premotor cortex, and DLPFC in HC. However, in patients with SCZ, since the natural frequency response itself was generally attenuated, there was no clear induction of site-specific natural frequencies by single-pulse TMS as in HC. Furthermore, lowered natural frequency in the prefrontal areas was related to the PANSS positive subscale scores as well as reaction time in the word memory task in patients with SCZ. Frantseva et al. administered single-pulse TMS and sham TMS over the left M1 in patients with SCZ and schizoaffective disorders and HC to analyze the time-domain, frequency-domain, and time-frequency-domain of TEP, separately [53]. The study demonstrated that the TMS-induced cortical activation in the gamma frequency band between 400 and 700 ms over the M1 was positively correlated with positive symptom severity in patients with SCZ. In contrast, the activation in theta and delta frequency bands at 200 ms after TMS was positively correlated with negative symptom severity in patients with SCZ. These results suggest that excessive gamma and theta/delta activations over the M1 may account for the underlying pathophysiology of positive and negative symptoms in SCZ. Moreover, it is proposed that excessive cortical activation can abnormally propagate to the remote areas of the cognitive domain, which may result in cognitive deficits due to impaired information processing in patients with SCZ. To test this, Canali et al. applied single-pulse TMS to the premotor cortex to investigate the natural frequency and its ERSP among patients with SCZ, bipolar disorder, major depressive disorder, and HC [56]. Specifically, ERSP was computed to quantify the response in the time-frequency domain. Natural frequency in the frontal area was significantly slower in each patient group compared with HC, while frontal natural frequencies were not significantly different among the patient groups. In addition, there was no correlation between natural frequency in the frontal area, PANSS scores, and medication dose in these patients. Similar to the natural frequency results, TMS-induced gamma oscillations were significantly slower in all the three diagnostic groups compared to HC, while there was no significant difference in the frequency of gamma oscillations over the PFC between the patient groups.

Ferrarelli et al. investigated the time-domain and frequency-domain of TEP in M1 applying single-pulse TMS over the left M1 in patients with first-episode psychosis (FEP) and HC [51]. Specifically, the global mean field power for the time domain was not significantly different between patients with FEP and HC. For the relative spectral power assessed for the frequency domain, patients with FEP showed a significantly decreased beta/low-gamma activities on TEP at the fronto-central area compared to HC. Furthermore, the lower cumulative spectral power was associated with worse scores on the scales of positive symptoms as well as negative symptoms in the PANSS. These results suggest that TMS-evoked fast oscillations over the fronto-central areas may be impaired from the time of onset. Andrews et al. applied single-pulse TMS to M1 when participants observed the other’s hand movements, which were designed to elicit mirror neuron system activity [52] and for which the facial affect recognition and theory of mind tasks were administered. The mu rhythm (8–13 Hz) was measured from the C3, Cz, and C4 EEG electrodes over the sensorimotor cortex. They found that patients with SCZ showed lower accuracy on the facial affect recognition and theory of mind tasks compared to HC. However, there were no significant differences in the degree of mu suppression and motor resonance between patients with SCZ and HC. These findings suggest that patients with SCZ may have an intact mirror neuron system.

#### 3.4.2. Paired-Pulse TMS Paradigm

LICI: The results are summarized in Table 2. SCZ showed significantly reduced LICI in the DLPFC [60], and it was correlated with working memory dysfunction [59]. Thus, the prefrontal aberrant GABA_B_ receptor-mediated dysfunction in SCZ may be part of the cause of working memory deficit in this disorder. Radhu et al. investigated LICI in the M1 and DLPFC in patients with SCZ, patients with bipolar disorder, and HC [60]. They assessed overall cortical inhibition in the frequency band from 1 to 50 Hz through the cluster mass tests between the groups. The LICI was significantly reduced in patients with SCZ compared with the other groups in the DLPFC not in M1, suggesting that LICI deficits in the DLPFC may be specific to the pathophysiology of SCZ. Lett et al. investigated the relationship between LICI in the DLPFC and glutamic acid decarboxylase I gene, a major determinant of GABA, in patients with SCZ [59]. They found that the variation in the glutamic acid decarboxylase I gene might play a pivotal role in the GABAergic inhibitory neurotransmission in the DLPFC and working memory performance in SCZ. In other words, working memory dysfunction may be attributable to the prefrontal aberrant GABA_B_ receptor-mediated function in patients with SCZ.

### 3.5. Connectivity Analyses

#### 3.5.1. Single-Pulse TMS Paradigm

The results are shown in Table 3. SCZ showed the defects in gamma cortical activity and connectivity over the frontal region [49,57]. Ferrarelli et al. applied single-pulse TMS to the parietal, M1, premotor, and DLPFC in patients with SCZ and HC [49]. They analyzed the significant current density (SCD) [62], which captures the amplitude of TMS-evoked cortical currents, indexing the cortical activity, as well as the significant current scattering (SCS) [62], which reflects the average distance of TMS-activated cortical sources, representing the cortical connectivity. They found that both SCD and SCS were most impaired in the DLPFC after single-pulse TMS in patients with SCZ compared with HC, but there was no difference in SCD or SCS over the parietal cortex and M1 after single-pulse TMS. There was a negative correlation between SCD and performance in episodic memory by the Penn Word memory delayed task, whereas higher SCS values were associated with a lower executive function assessed by the Penn Conditional Exclusion test. Those findings indicate that the defects in cortical activity and connectivity of the DLPFC may underlie the pathophysiology of cognitive impairments in patients with SCZ.

One study administered single-pulse TMS to the premotor cortex to analyze amplitude measures using global mean field power and ERSP as well as synchronization measures using intertrial coherence of TEP and source localization in patients with SCZ and HC [57]. Patients with SCZ showed significantly decreased amplitude and synchronization of TMS-evoked gamma oscillations particularly in the frontocentral area during the 100 ms after TMS pulse compared with HC. In the source modeling analysis, patients with SCZ presented with a slow propagation of TMS-evoked gamma oscillations from the bilateral premotor cortex to the bilateral M1 along with the midline, whereas HC showed the gamma propagation from the bilateral premotor cortex to the right sensorimotor, left anterior premotor, and left sensorimotor areas. Given the findings in previous studies that gamma oscillations occur in the corticothalamic loop [63,64], the above findings suggest that there may be intrinsic dysfunctions in the frontal thalamocortical circuits in patients with SCZ [50].

#### 3.5.2. Paired-Pulse TMS Paradigm

LICI: The results are summarized in Table 3. SCZ showed significant deficits in cortical inhibition as well as inhibitory gamma modulation in the DLPFC during the LICI paradigm [54,55]. Farzan et al. conducted the LICI paradigm over the left M1 and DLPFC in patients with SCZ and major depressive disorder, and HC using active and sham stimulation conditions [54]. They analyzed the modulatory effect of LICI on cortical oscillations across the five frequency bands as follows: δ (1–3 Hz), θ (4–7 Hz), α (8–12 Hz), β (12.5–28 Hz), and γ (30–50 Hz). They found that patients with SCZ had significantly reduced cortical inhibition and induction of gamma oscillations by TMS-EEG neurophysiology in the DLPFC but not in M1 compared with the other groups. Radhu et al. measured LICI from the DLPFC in patients with SCZ or schizoaffective disorder, patients with obsessive-compulsive disorder, unaffected first-degree relatives of these patients, and HC [55]. They analyzed time-frequency decomposition to compare the cortical inhibitory function among these groups. As a result, they found that the degree of cortical inhibition was significantly decreased in patients with SCZ compared to HC. In addition, there was no significant difference in the degree of cortical inhibition between HC and first-degree relatives of patients with SCZ. Therefore, the deficits of LICI in the DLPFC may represent one of the diagnostic biomarkers of SCZ.

### 3.6. Risk of Bias

Out of 14 studies, 11 (79%) showed a low risk of bias for the participant selection, 10 (71%) showed a low risk of bias for the confounding variables, and all of the included studies showed a low risk of bias for the measurement of exposure, blinding of outcome assessment, and incomplete outcome data. On the other hand, 10 studies (71%) were judged to be unclear for the selective outcome reporting because the details of most research protocols were not specified in the articles.

## 4. Discussion

### 4.1. Summary of This Review

The present systematic review found that patients with SCZ had oscillatory abnormalities, especially in the prefrontal cortex. Indeed, for the DLPFC, patients with SCZ showed inhibitory (i.e., SICI and LICI) and facilitatory (i.e., ICF) dysfunctions [55,58,60]; however, for M1, there was no significant dysfunction in cortical inhibition (i.e., LICI) or TMS-induced gamma oscillations [54,60]. On the other hand, a previous study assessing I-wave facilitation of SCZ with TMS-EMG reported that there may be some deficit in cortical inhibition in M1 [65]. Furthermore, the other study reported that single-pulse TMS to M1 in patients with SCZ showed excessive gamma oscillations in the M1 region in the 400–700 ms post-stimulus interval [53]. Thus, further studies are needed to confirm whether the impairment of cortical inhibition of SCZ also extends to M1. Moreover, this review found reduced connectivity between the premotor cortex and prefrontal cortex [49] and reduced TMS-induced gamma oscillations in the fronto-central regions [57] in patients with SCZ. In addition, TMS-induced functional cortical conductivity in the gamma band was positively related to positive symptoms, while the functional cortical conductivity in the theta and delta bands was positively related to negative symptoms in SCZ [53]. Moreover, cholinergic dysfunction in the DLPFC, as indexed by SAI, was associated with cognitive impairment in SCZ [41].

### 4.2. Evidence to Support the E/I Imbalance Hypothesis in SCZ

The E/I imbalance hypothesis in SCZ postulates that an imbalance between excitation and inhibition in neural circuits would be involved in the pathophysiology of SCZ, which may be related to clinical symptoms and cognitive deficits in patients with SCZ. Numerous studies have reported abnormalities in the excitatory function of the glutamatergic system and the inhibitory function of the GABAergic system in patients with SCZ [66,67,68,69]. Based on the E/I imbalance hypothesis, it would be anticipated that patients with SCZ may have altered GABAergic function as indexed by SICI and LICI paradigms as well as increased or decreased glutamatergic function as indexed by ICF paradigm depending on the clinical condition and stage of this disorder.

### 4.3. Evidence to Support the GABA Hypothesis in SCZ

Previous studies have reported decreased SICI and LICI in the DLPFC of SCZ, suggesting that GABA_A_ receptor- and GABA_B_ receptor-mediated dysfunctions in the DLPFC are pathological features of SCZ [58,60]. Furthermore, previous studies showed that clozapine, an atypical antipsychotic medication, has the binding potential to the GABA_B_ receptor and acts as a modulator for the receptor [70,71]. Indeed, Daskalakis et al. reported that clozapine treatment was associated with increased cortical inhibition in patients with SCZ, which might be exerted by the potentiation of cortical GABA_B_ receptor-mediated inhibitory neurotransmission [20,72]. Likewise, other studies that examined the TMS-EMG on patients with SCZ indicated that SCZ had significantly decreased SICI compared with HC even after controlling for age and medications [45]. In addition, postmortem studies noted that there was decreased GABA levels in patients with SCZ [73].

### 4.4. Evidence to Support the Glutamate Hypothesis in SCZ

On the other hand, one study has shown that patients with SCZ have reduced ICF on the DLPFC, suggesting reduced function via glutamatergic NMDA receptors [58], supporting the glutamate hypothesis. For example, phencyclidine, a prototype of noncompetitive NMDA receptor antagonist, induces psychotic symptoms, thought disorder, blunted affect, and cognitive impairments in healthy individuals [74]. However, for the ICF, a previous meta-analysis study revealed that there was no significant difference in ICF between patients with SCZ and HC after controlling for age and medications [45]. A recent meta-analysis of ^1^H-MRS studies noted that there were no significant changes in glutamate levels in the DLPFC in patients with SCZ [13,75]. However, the present study showed decreased ICF in patients with SCZ as measured by TMS-EEG that come from one study [58]. Thus, our finding warrants further studies to confirm the present study.

### 4.5. Potential Evidence to Support the Cholinergic Hypothesis in SCZ

In addition, the decrease in SAI in the DLPFC of SCZ reported by Noda et al. indicates that the cholinergic function of the region may be reduced in SCZ [41], supporting the cholinergic hypothesis [76]. However, in the TMS-EEG experiments of Noda et al. at that time, noise masking methods such as white noise were not used to suppress the auditory evoked potential (AEP) to TMS clicks. Therefore, it is possible that there was contamination of the AEP by the TMS click sounds in the N100-P180 complex of TEP [77]. Therefore, it is necessary to confirm the reproducibility of the results of this study using a more accurate experimental method in the future. In addition, cholinergic receptors (α7-nicotinic acetylcholine receptors, α7-nAChRs) have recently been considered a potential therapeutic target for SCZ as well as other cognitive disorders without causing adverse effects [78]. Indeed, this notion is supported by multiple lines of evidence, ranging from genetic studies to behavioral studies. For example, a postmortem brain study has indicated deficits of α7-nAChRs in the DLPFC and hippocampus of patients with SCZ [79]. Furthermore, such deficits are thought to contribute to abnormalities in smooth pursuit eye movements, sustained attention, and other cognitive domains in patients with SCZ [80].

### 4.6. Abnormalities of TMS-Induced Gamma Oscillations in SCZ

The present review also noted that TMS-evoked gamma oscillations in the frontocentral area were significantly reduced in patients with SCZ, which was also associated with reduced effective connectivity in related regions. Furthermore, since GABAergic function in the cerebral cortex is reduced in SCZ, the E/I balance is also likely to be naturally impaired [81], which may result in reduced natural frequency, especially in the prefrontal cortex [50]. However, on the other hand, studies that assessed frontal natural frequency by TMS-EEG in patients with major depressive disorder and bipolar disorder have also shown a decreased natural frequency, suggesting that this finding may be a pathophysiology that is at least in part shared among the major psychiatric disorders [56]. Specifically, since gamma band activity in the DLPFC is supposed to be related to higher-order cortical processing [82,83], these findings may represent neural bases that contribute to the pathophysiology of SCZ [84,85]. Taken together, these findings suggest that patients with SCZ may involve dysfunction of the frontal-thalamocortical circuits necessary to execute appropriate information processing [50,57]. Moreover, the similar abnormal findings observed in the PFC can also be found in M1 in patients with SCZ, which may be due to the effects of the corresponding abnormal network resonance properties on M1 [53]. Moreover, Ferrarelli et al. measured cortical oscillatory responses with single-pulse TMS targeting the left frontal, parietal, and occipital cortices at rest, and they showed that different cortical networks could be characterized by different oscillatory activities [57]. Specifically, they showed that each cortical region may respond at a characteristic frequency, termed the natural frequency, described above. In addition, the study by Ferrarelli et al. also showed that the topography of TMS-evoked oscillatory activity was changed corresponding to the site of stimulation. Moreover, the pattern of topography corresponding to each stimulation site hardly overlaps between target sites, suggesting that functionally separated networks in HC oscillate at different frequencies at rest [86,87]. On the other hand, Andrew et al. first investigated the activity of the mirror system in response to behavioral observations in SCZ using TMS-EEG and found that mu-suppression and motor resonance were related. In addition, the study demonstrated that the mirror system was intact in the stable SCZ, suggesting that other neural substrates may be involved in the social cognitive deficits in SCZ [52].

### 4.7. Insights from This Systematic Review

Ferrarelli et al. showed that TMS-evoked gamma oscillations in the fronto-central areas occurring within 100 ms after single-pulse TMS were significantly reduced in SCZ, and amplitude and phase synchronization in the same gamma oscillations were also significantly reduced, and source modeling analysis showed that TMS-evoked EEG propagation was found to be restricted to the TMS stimulation site compared to HC [57]. On the other hand, a study by Frantseva et al. observed excessive gamma oscillations at the M1 400–700 ms after single-pulse TMS over the M1 in patients with SCZ [53]. In the same study, functional cortical conductivity of TMS-induced gamma activity was positively correlated with positive symptoms, while functional cortical conductivity of theta and delta bands was positively correlated with negative symptoms in patients with SCZ [53]. In addition, a SICI study by Noda et al. [58] and LICI studies by Radhu et al. [55,60] have shown that patients with SCZ had decreased GABA receptor-mediated function as indexed by SICI and LICI paradigms as well as decreased glutamatergic NMDA receptor-mediated function as indexed by ICF paradigm in the DLPFC. Furthermore, studies by Farzan et al. [54] and Radhu et al. [55] have shown that LICI paradigm that applied to the DLPFC in patients with SCZ induced significantly lower power of gamma oscillations at the same region within 100 ms after TMS when compared with HC, whereas those studies have shown that there was no significant reduction of GABAergic inhibitory function or gamma oscillation activity in patients with SCZ when applying the LICI paradigm to M1 [54,60]. Although several studies using measurement modalities other than TMS neurophysiology [66,67,68,69] have already reported abnormalities in glutamatergic excitatory function and GABAergic inhibitory function in patients with SCZ, the present systematic review of TMS-EEG neurophysiology indicates that GABAergic function, mainly in the frontal region, could be decreased in patients with SCZ [81], and E/I balance in the prefrontal and frontal-thalamic cortical responses is likely to be impaired [50]. In particular, since reduced TMS-evoked gamma oscillations in the DLPFC of patients with SCZ are associated with higher-order information processing deficits [82,83], these findings may explain the pathophysiology and neural basis of cognitive dysfunction in SCZ [57,84,85]. Therefore, exactly as the E/I imbalance hypothesis proposes, the imbalance between excitation and inhibition in neural circuits may be involved in the pathophysiology of SCZ, which may be related to the clinical symptoms and cognitive deficits of this disorder.

### 4.8. Limitations of This Review and TMS-EEG Study in General

Fourteen research papers were extracted for this review, all of which are TMS-EEG studies in schizophrenia. However, the research objectives and methodologies of each paper are different, and it is currently difficult to discuss, interpret, and integrate the results of each study in a truly systematic way. Therefore, it is necessary to verify the reproducibility of the results of each study and establish a more reliable methodology through further research in this area.

Combined TMS-EEG is a non-invasive and useful evaluation method for neuropsychiatric disorders; however, there is a limitation on the principle to this modality that it cannot directly measure the neuron activity itself. Since there were only 14 studies in the current review that met the inclusion criteria, it must be said that at present, we can report preliminarily results in this discipline. For example, there is only one TMS-EEG study that examined the cholinergic system in schizophrenic [41], and more studies with larger sample sizes are needed to establish sufficient evidence. Other common problems of TMS-EEG are as follows: muscle activities, TMS-induced cranial reflexes, and somatosensory and auditory evoked potentials can easily contaminate TMS-EEG responses as artifacts [2]. In particular, the detection of EEG gamma rhythm can be affected by various artifacts derived from TMS [88]. In addition, since the effects of psychotropic medications on EEG activities cannot be ignored, a study design that controls medication in patients with SCZ is also important in TMS-EEG research [89]. Alternatively, at least, elaborated analyses that control the effect of the medication is required. Furthermore, most of the previous TMS-EEG studies have been conducted by various recording systems, different experimental methods, and unique analysis methods. Thus, these methodological differences in the included studies make it difficult to interpret the results. Hence, future research applying more standardized experimental methods with larger sample sizes in a transdiagnostic approach will help improve the quality of TMS-EEG study [89] and further such sophisticated TMS-EEG research will be important to replicate and confirm the currently available findings of this special modality [17]. In addition, previous TMS-EEG studies have reported TMS neurophysiological findings in patients with SCZ at various clinical stages, but it remains unclear at which clinical stage of SCZ these abnormal neurophysiological findings occur and how they progress. Therefore, to address the above issues, future TMS-EEG studies must use standardized experimental and analytical methods to evaluate longitudinal changes in neurophysiological findings from the pre-onset, prodromal, acute, and chronic stages of SCZ.

## 5. Conclusions

The findings of this systematic review support, at least in part, several hypotheses that explain the pathophysiological bases of SCZ, including the E/I imbalance hypothesis discussed in the introduction. Furthermore, the present systematic review demonstrated the usefulness of combined TMS-EEG to identify the neurophysiological biomarkers to better understand the neural bases of this disorder. Moreover, this modality can be applied to develop objective diagnostics of this disorder, facilitate the prognostication of clinical symptoms, and improve therapeutic strategies for patients with SCZ.

## Figures and Tables

**Table 1:** TMS-evoked potentials (TEP) analyses.

**(A)**	***Single-Pulse TMS Paradigm***												
**Authors, Year**	**Patient Group**	**Age (Mean ± SD)**	**Number of Subjects (Female)**	**Clinical Severity (Mean ± SD)**	**Medication**	**Control Group**	**Age (Mean ± SD)**	**Number of Subjects (Female)**	**Stimulation Parameters**	**Areas of Stimulation**	**Cognitive Measures**	**Analyses**	**Neurophysiological Findings**
Levit-Binnun et al., 2009	SCZ	38 ± 8	8(0)	PANSS	Five patients were on atypical antipsychotics (ziprasidone) and two were on typical antipsychotics (haloperidol and fluphenazine: mean dose equivalent of 415 mg chlorpromazine).	HC	29 ± 10	6(3)	Single-pulse TMS Sham	Over the Cz electrode	N/A	TMS-evoked potential (TEP) analysis Amplitude Latency	In HCs, clear frontal negativity and parietal positivity were observed at 29 ms after TMS stimulation, but in SCZ, there was no frontal negativity and parietal positivity was greatly reduced.
Noda et al., 2018	Chronic SCZ	41 ± 10	12(4)	PANSS total: 50 ± 6.2 PANSS negative: 12 ± 3.4 PANSS positive: 11 ± 3.0 PANSS general: 24 ± 2.8	Patients were taking chlorpromazine equivalent dose (330 ± 290 mg/day) of antipsychotics.	HC	39 ± 12	12(6)	Short-latency afferent inhibition (SAI)	Left primary motor cortex (M1) Dorsolateral prefrontal cortex (DLPFC)	Wechsler Test of Adult Reading Letter-Number Span Test Hopkins Verbal Learning Test Trail Making Test	TMS-evoked potential (TEP) analysis Amplitude of components	Patients with SCZ had significantly smaller modulation of N100 by DLPFC-SAI compared to HC, suggesting impaired cholinergic neurophysiological function in DLPFC. Furthermore, reduced DLPFC-SAI correlated with executive dysfunction as measured by TMT.
**(B)**	***Paired-Pulse TMS Paradigm***												
**Authors, Year**	**Patient Group**	**Age (Mean ± SD)**	**Number of Subjects (Female)**	**Clinical Severity (Mean ± SD)**	**Medication**	**Control Group**	**Age (Mean ± SD)**	**Number of Subjects (Female)**	**Stimulation Parameters**	**Areas of Stimulation**	**Cognitive/Clinical Measures**	**Analyses**	**Neurophysiological Findings**
Noda et al., 2017	SCZ	41 ± 10	12(4)	PANSS total: 50 ± 6.2 PANSS negative: 12 ± 3.4 PANSS positive: 11 ± 3.0 PANSS general: 24 ± 2.8	Patients were taking chlorpromazine equivalent dose (330 ± 290 mg/day) of antipsychotics.	HC	39 ± 12	12(6)	Short interval intracortical inhibition (SICI) Intracortical facilitation (ICF)	Left dorsolateral prefrontal cortex (DLPFC)	Wechsler Test of Adult Reading Letter-Number Span Test Trail Making Test Hopkins Verbal Learning Test	TMS-evoked potential (TEP) analysis Amplitude of components Frequency band powers Time-frequency analysis	Patients with SCZ showed reduced inhibition in TEP P60 by DLPFC-SICI compared with HC, which was correlated with the longest span of the LNST. Further, patients with SCZ showed reduced facilitation in TEP P60 and N100 by DLPFC-ICF compared with HC, which were correlated with the total score of the PANSS.

**Table 2:** Time-frequency analyses.

**(A)**	***Single-Pulse TMS Paradigm***												
**Authors, Year**	**Patient Group**	**Age (Mean ± SD)**	**Number of Subjects (Female)**	**Clinical Severity (Mean ± SD)**	**Medication**	**Control Group**	**Age (Mean ± SD)**	**Number of Subjects (Female)**	**Stimulation Parameters**	**Areas of Stimulation**	**Cognitive Measures**	**Analyses**	**Neurophysiological Findings**
Ferrarelli et al., 2012	SCZ	33 ± 6.2	20(7)	PANSS general: 39 ± 11 PANSS negative: 22 ± 6.0 PANSS positive: 18 ± 6.3	Eighteen patients were taking second-generation antipsychotics while two were on first-generation antipsychotics.	HC	32 ± 7.8	20(4)	Single-pulse	Posterior parietal cortex Motor cortex Premotor cortex Prefrontal cortex	The word memory Penn Word Recognition Test The facial memory Penn Facial Memory Test	Time-Frequency Analysis Event-related spectra perturbation (ERSP) Intertrial coherence (ITC)	In patients with SCZ, the natural frequency response was generally attenuated compared with HC when single-pulse TMS was applied to the prefrontal cortex. Further, the lowered natural frequency in the prefrontal areas in SCZ was related to the PANSS positive scores and reaction time in the word memory task.
Frantseva et al., 2014	SCZ or schizoaffective disorder	37 ± 10	16(4)	PANSS total: 65 ± 18 PANSS negative: 18 ± 6.1 PANSS positive: 16 ± 4.3 PANSS global: 30 ± 8.6	Fourteen patients with schizophrenia were treated with antipsychotic medications (clozapine: n = 6, mean dose 400 ± 55 mg/day; risperidone: n = 3, mean dose 3.2 ± 2.5 mg; haloperidol: n = 2, mean dose 2.0 ± 1.4 mg; quetiapine: n = 1, 100 mg; perphenazine: n = 1, 16 mg; olanzapine: n = 1, 7.5 mg) and two patients did not take any psychotropic medications.	HC	36 ± 7.9	16(5)	Single-pulse (single monophasic TMS pulse) Sham	Left motor cortex	N/A	Time-domain Analysis Frequency-domain Analysis Time-Frequency Analysis	TMS-induced cortical activation in the gamma band between 400 and 700 ms at the M1 was positively correlated with positive symptoms in patients with SCZ. In contrast, the activation in theta and delta bands at 200 ms after TMS was positively correlated with negative symptoms in patients with SCZ.
Canali et al., 2015	Chronic undifferentiated SCZ	38 ± 9	12(3)	PANSS general: 37 ± 5 PANSS negative: 18 ± 4 PANSS positive: 18 ± 4	All patients were taking antipsychotics (typical antipsychotics: n = 5; atypical antipsychotics: n = 7).	HC	39 ± 15	12(7)	Single-pulse	Premotor cortex	N/A	Time-Frequency analysis Event-related spectral perturbation (ERSP)	Natural frequency in the frontal cortex was significantly slower in patients with bipolar disorder (20 ± 3.7 Hz), major depressive disorder (19 ± 5.0 Hz), and SCZ (20 ± 4.2 Hz) than HC (27 ± 3.2 Hz). However, frontal natural frequencies among the patient groups (i.e., bipolar disorder, major depressive disorder, and SCZ) were not significantly different. There was no correlation among natural frequency in the frontal area, PANSS scores, and medication dose in these populations.
Ferrarelli et al., 2019	First-episode psychosis(FEP)	23 ± 5.2	16(4)	Scale for the assessment of positive symptoms (SAPS): 18 ± 13 Scale for the assessment of negative symptoms scores (SANS): 31 ± 12	Nine FEP patients were antipsychotic naïve and seven patients were taking antipsychotics less than 1 month.	HC	23 ± 6.3	11(3)	Single-pulse	Left primary motor cortex (M1)	N/A	Time-Frequency analysis Time domain: The global mean field power (GMFP) Frequency domain: Relative spectral power (RSP)/Cumulated RSP (cRSP)	GMFP for the time domain was not significantly different between patients with FEP and HC. When RSP was assessed for the frequency domain, patients with FEP showed a significantly decreased beta/low-gamma TEP activities at the fronto-central area relative to HC. The lower RSP was associated with both worse scores on the SAPS and the SANS. TMS-evoked fast oscillations over the fronto-central areas were impaired from the time of onset, suggesting that these deficits may be related to the clinical symptoms.
Andrews et al., 2015	SCZ or schizoaffective disorder	44 ± 11	19	PANSS general: 34 ± 8.1 PANSS negative: 16 ± 5.5 PANSS positive: 15 ± 6.1	Patients were taking chlorpromazine equivalent dose (67~1307 mg/day) of antipsychotics.	HC	38 ± 13	19	Single-pulse TMS during the observation of hand movements designed to elicit mirror system activity	Primary motor cortex (M1)	NimStim Static Face Task Cognitive and Affective Mental Inference Task (Inference Task)	Frequency analysis mu rhythm (8–13 Hz) TMS induced motor-evoked potentials (MEPs)	Patients with SCZ showed lower accuracy on the facial affect recognition and theory of mind tasks than HC. No significant differences in the degree of mu suppression and motor resonance between the patients with SCZ and HC.
**(B)**	***Paired-Pulse TMS Paradigm***												
**Authors, Year**	**Patient Group**	**Age (Mean ± SD)**	**Number of Subjects (Female)**	**Clinical Severity (Mean ± SD)**	**Medication**	**Control Group**	**Age (Mean ± SD)**	**Number of Subjects (Female)**	**Stimulation Parameters**	**Areas of Stimulation**	**Cognitive Measures**	**Analyses**	**Neurophysiological Findings**
Radhu et al., 2015	SCZ	36	38(13)	Brief Psychiatric Rating Scale (BPRS)	All patients were treated with antipsychotics.	HC	34	46(23)	Long-Interval Cortical Inhibition (LICI)	Left motor cortex Dorsolateral prefrontal cortex (DLPFC)	N/A	Time-Frequency analysis	LICI was significantly reduced in patients with SCZ compared with other groups in the DLPFC not in M1.
Lett et al., 2016	SCZ or schizoaffective disorder	35 ± 10	23(5)	N/A	Not reported	HC	35 ± 11	33(18)	Long-Interval Cortical Inhibition (LICI)	Dorsolateral prefrontal cortex (DLPFC)	IQ Wechsler Test of Adult ReadingWorking memory Letter-number sequencing task Digit-span forward taskSelective attention Stroop Neuropsychological Screening Test	Time-Frequency analysis Cluster-Based analysis	Variation of the GAD1 gene in patients with SCZ may play a pivotal role in GABA(B)ergic inhibitory neurotransmission and working memory performance in the DLPFC.

**Table 3:** Connectivity analyses.

**(A)**	***Single-Pulse TMS Paradigm***												
**Authors, Year**	**Patient Group**	**Age (Mean ± SD)**	**Number of Subjects (Female)**	**Clinical Severity (Mean ± SD)**	**Medication**	**Control Group**	**Age (Mean ± SD)**	**Number of Subjects (female)**	**Stimulation Parameters**	**Areas of Stimulation**	**Cognitive Measures**	**Analyses**	**Neurophysiological Findings**
Ferrarelli et al., 2015	SCZ	33 ± 6.2	20(7)	PANSS general: 39 ± 11 PANSS negative: 22 ± 5.8 PANSS positive: 18 ± 6.3	Patients were taking chlorpromazine equivalent dose (314 ± 129 mg/day) of antipsychotics.	HC	32 ± 7.8	20(4)	Single-pulse TMS	Prefrontal cortex (PFC) Premotor cortex Motor cortex Parietal cortex	Episodic Memory Word Memory Delayed Executive Function Penn Conditional Exclusion Test	TMS-evoked potential (TEP) analysis Significant current density (SCD) Connectivity analysis Significant current scattering (SCS)	Both SCD and SCS were most impaired in the DLPFC after single-pulse TMS in patients with SCZ compared with HC. No difference in SCD and SCS were observed in the parietal cortex and M1 after single-pulse TMS. SCD and performance in episodic memory were negatively correlated, whereas higher SCS values were associated with a lower executive function.
Ferrarelli et al., 2008	SCZ	34 ± 8.0	16(3)	PANSS	N/A	HC	35 ± 7.0	14(3)	Single-pulse TMS	Right premotor cortex	N/A	TMS-evoked potential (TEP) analysis Amplitude The global mean field power (GMFP) Event-related spectral perturbation phase Connectivity analysis	Patients with SCZ indicated significantly decreased amplitude and synchronization of TMS-evoked gamma oscillations particularly in the frontocentral region during the 100 ms after TMS pulse compared with HC. In the source modeling analysis, cortical propagation of TMS-evoked gamma oscillations was more localized compared with HC.
**(B)**	***Paired-Pulse TMS Paradigm***												
**Authors, Year**	**Patient Group**	**Age (Mean ± SD)**	**Number of Subjects (Female)**	**Clinical Severity (Mean ± SD)**	**Medication**	**Control Group**	**Age (Mean ± SD)**	**Number of Subjects (Female)**	**Stimulation Parameters**	**Areas of Stimulation**	**Cognitive Measures**	**Analyses**	**Neurophysiological Findings**
Farzan et al., 2010	SCZ	38 ± 10	14(4)	PANSS total: 66 ± 18 PANSS negative: 18 ± 6.3 PANSS positive: 17 ± 4.4 PANSS global: 31 ± 9.2	Two patients were unmedicated (one medication-naive; one medication-free for 6 months) and 12 patients were on medication (n = 5, 390.0 ± 54.8 mg clozapine; n = 3, 3.2 ± 2.5 mg risperidone; n = 2, 2 ± 1.4 mg haloperidol; n-1, 100 mg of quetiapine; n = 1, 16 mg perphenazine)	HC	37 ± 7.6	14(5)	Long-Interval Cortical Inhibition (LICI) Sham	Left motor cortex Dorsolateral prefrontal cortex (DLPFC)	N/A	Time-Frequency analysis	Patients with SCZ had significant deficits of cortical inhibition and inhibitory modulation of gamma oscillations in the DLPFC but not in M1 compared with the other groups.
Radhu et al., 2017	(a) SCZ or schizoaffective disorder (b) First-degree relatives of patients with SCZ	(a) 30 (b) 54	(a) 19(9) (b) 30(17)	Schizotypal Personality Questionnaire The 24-construct Brief Psychiatric Rating Scale	Patients were taking clozapine (150~475 mg/day).	HC	33	49(25)	Long-Interval Cortical Inhibition (LICI)	Motor cortex Dorsolateral prefrontal cortex (DLPFC)	N/A	Time-Frequency analysis	The degree of cortical inhibition as indexed by LICI was significantly decreased in patients with SCZ compared to HC. Further, no significant difference in the degree of cortical inhibition between HC and first-degree relatives of patients with SCZ.

## Data Availability

Data sharing not applicable.

## Abbreviations

DLPFC: dorsolateral prefrontal cortex; EEG: electroencephalograph; E/I: excitatory and inhibitory; ERSP: event-related spectral perturbation; FEP: first-episode psychosis; GABA: gamma-aminobutyric acid; HC: healthy control; ^1^H-MRS: proton magnetic resonance spectroscopy; ICF: intracortical facilitation; ISI: interstimulus interval; LICI: long-interval cortical inhibition; MEP: motor-evoked potential; M1: primary motor cortex; NMDA: N-methyl-d-aspartate; PANSS: positive and negative syndrome scale; PFC: prefrontal cortex; SAI: short-latency afferent inhibition; SCD: significant current density; SCS: significant current scattering; SCZ: schizophrenia; SICI: short-interval intracortical inhibition; TEP: TMS-evoked potential; TMS: transcranial magnetic stimulation; Preferred Reporting Items for Systematic Reviews and Meta-Analyses: PRISMA.
